# Blood Flow Restriction in Athletic Populations—Part 3: Applications for Cardiovascular Conditioning, Performance Priming, and Clinical Management

**DOI:** 10.3390/jfmk11040423

**Published:** 2026-10-15

**Authors:** Chris Gaviglio, Christian J. Cook, Stephen P. Bird

**Affiliations:** 1 School of Health, Psychological and Medical Sciences, University of Southern Queensland, Ipswich 4305, Australia; stephen.bird@unisq.edu.au; 2 Institute for Health, University of Southern Queensland, Ipswich 4305, Australia; 3 Biomedical Discipline, School of Science and Technology, University of New England, Armidale 2350, Australia

**Keywords:** blood flow restriction, occlusion training, aerobic conditioning, ischemic preconditioning, sport-specific training, rehabilitation, priming

## Abstract

**Background:** The application of blood flow restriction (BFR) training extends beyond traditional resistance, with targeted use in cardiovascular conditioning, performance priming, and clinical applications relevant to athletic populations. Part 3, the final paper of this series, focuses on the application of blood flow restriction (BFR) during cardiovascular exercise (BFR-CV), ischemic preconditioning (IPC), and therapeutic applications, including the management of joint pain, tendinopathy, and bone health. **Methods:** A narrative review of research examining BFR applications for cardiovascular conditioning, performance priming, and clinical management was conducted using structured searches of PubMed/MEDLINE, SPORTDiscus, Scopus, and Web of Science, supplemented by targeted literature searching and reference-list screening through May 2026. Search terms encompassed BFR and occlusion training alongside cardiovascular and aerobic exercise, anaerobic and interval training, sprint and sport-specific conditioning, ischemic preconditioning and intra-conditioning, pain and tendinopathy, and bone health. Evidence from trained and athletic populations was prioritized, with relevant clinical and mechanistic studies included where athlete-specific evidence was limited or unavailable. Findings were synthesized narratively across conditioning, IPC/IIC, pain-management, and bone-health applications. **Results:** Low-intensity BFR-CV (40–50% VO_2max_ with 40–80% arterial occlusion pressure) may enhance aerobic capacity and muscular endurance in trained athletes while minimizing mechanical loading. Sport-specific BFR applications during soccer drills, basketball activities, and small-sided games have been associated with improvements in repeated-sprint ability and sport-specific endurance without impairing speed or power. IPC may produce small, context-dependent improvements (1–3%) in short-duration, high-intensity performance and may support recovery between repeated competitive bouts. Low-load BFR training (20–30% 1RM) may reduce joint and tendon pain while maintaining muscle strength and mass during rehabilitation. Evidence for bone health remains preliminary, with BFR showing potential to influence bone-turnover markers and preserve musculoskeletal function during periods of restricted osteogenic loading. **Conclusions:** BFR represents a versatile modality for conditioning, performance optimization, and athlete care when mechanical load or training time is constrained.

## 1. Introduction

Blood flow restriction (BFR) training has emerged as a versatile performance tool that extends well beyond resistance exercise, encompassing cardiovascular-based conditioning, pre-competition priming, and therapeutic uses. In this three-part series, Parts 1 and 2 established BFR’s physiological foundations and resistance training applications, while Part 3 examines BFR applications in conditioning and therapeutic domains, which remain less systematically defined. Although much of the available evidence for BFR during aerobic or sport-specific conditioning is derived from apparently healthy, moderately trained populations, emerging studies in athletic populations demonstrate meaningful adaptations in aerobic capacity, repeated-sprint performance, and sport-specific endurance. Beyond these conditioning applications, growing interest also surrounds the use of BFR as an adjunct for managing joint pain, tendon loading, and bone-stress-related injuries [1,2,3,4,5,6]. Ischemic preconditioning (IPC), which involves short cycles of occlusion and reperfusion applied before or between efforts, represents another emerging application with potential for pre-competition or intra-session priming and recovery [7,8,9,10,11,12]. Understanding such applications is particularly relevant given the increasing demands on athlete availability [13], the need to maintain fitness during injury rehabilitation, and the growing emphasis on training load management in high-performance environments. Collectively, the aim of this paper is to provide an overview of the BFR literature specifically related to cardiovascular conditioning, performance priming, and therapeutic applications in clinical management of athletic populations, therefore providing evidence-informed guidance for practitioners.

**Figure 1:**
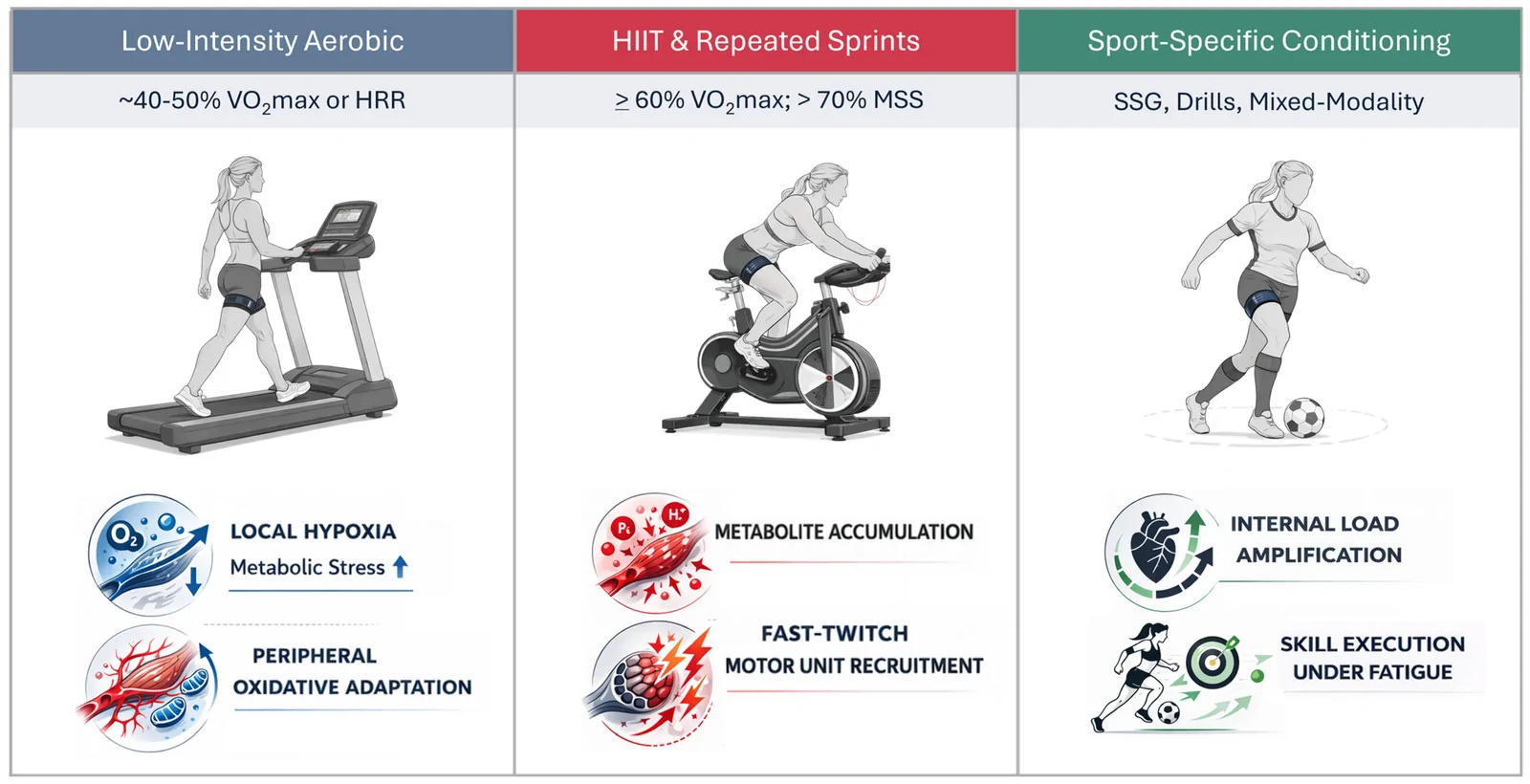
Conceptual overview of blood flow restriction (BFR) applications across cardiovascular and sport-specific conditioning, highlighting modality-dependent mechanistic and performance-related effects. Abbreviations: VO_2max_, maximal oxygen uptake; ↑, increase; ↓, decrease; HRR, heart rate reserve; MSS, maximal sprint speed; SSG, small-sided game; HIIT, high-intensity interval training.

Blood flow restriction during cardiovascular exercise (BFR-CV) has been extensively studied in clinical and general populations, demonstrating improvements in cardiorespiratory function [14,15], exercise capacity [16,17], muscle strength and hypertrophy [15,18,19,20]. However, the relevance of these findings to highly trained athletes requires careful consideration, as baseline fitness levels, training status, and performance demands differ substantially. BFR-CV protocols typically employ low intensities (approximately 40% VO_2max_ or 45% heart rate reserve) [15,17,21], though higher intensities (>60%VO_2max_) have also demonstrated training effects [22,23]. The following sections examine low-intensity aerobic exercise, high-intensity and sport-specific applications, and practical programming considerations for each modality. The subsequent sections progress from low-intensity BFR-CV evidence through higher-intensity and sport-specific protocols, before transitioning to ischemic preconditioning (IPC) and clinical applications. Figure 1 provides a conceptual overview of the application of BFR-CV across the cardiovascular training continuum.

Throughout this review, practical recommendations are derived from the characteristics and outcomes of the available literature and are supplemented, where required, by author interpretation to support translation into practice. Where specific numerical thresholds, decision criteria, or session-integration strategies have not been directly evaluated in published studies, these should be interpreted as pragmatic, evidence-informed recommendations rather than independently validated cut-offs.

## 2. Methods

### 2.1. Search Strategy

A narrative review of research examining BFR applications for cardiovascular conditioning, performance priming, and clinical management in trained and athletic populations was conducted. A comprehensive literature search of PubMed/MEDLINE, SPORTDiscus, Scopus, and Web of Science was performed using combinations of search terms relevant to the major application areas examined within this review. Search syntax was modified where required to accommodate database-specific indexing and terminology. The literature search included studies available through May 2026.

For cardiovascular, anaerobic, and sport-specific conditioning, searches included combinations of: (“blood flow restriction” OR “BFR” OR “occlusion training” OR “KAATSU”) AND (“aerobic exercise” OR “cardiovascular exercise” OR “endurance” OR “cycling” OR “running” OR “rowing” OR “swimming” OR “sprint” OR “repeated sprint” OR “interval training” OR “HIIT” OR “sprint interval training” OR “anaerobic” OR “conditioning” OR “sport-specific” OR “small-sided games”) AND (“athletes” OR “athletic” OR “trained” OR “elite” OR “sport”).

To capture the literature examining performance priming, recovery, and intra-session occlusion strategies, additional searches included combinations of: (“ischemic preconditioning” OR “ischaemic preconditioning” OR “IPC” OR “ischemic conditioning” OR “ischaemic conditioning” OR “ischemic intra-conditioning” OR “ischaemic intra-conditioning” OR “post-exercise blood flow restriction”) AND (“exercise” OR “performance” OR “recovery” OR “training” OR “sport” OR “athlete”).

For joint and tendon pain and bone-health applications, additional searches included combinations of: (“blood flow restriction” OR “BFR” OR “occlusion training”) AND (“pain” OR “hypoalgesia” OR “analgesia” OR “tendinopathy” OR “patellofemoral” OR “low back pain” OR “rehabilitation” OR “bone” OR “bone mineral density” OR “bone turnover” OR “bone stress injury” OR “stress fracture” OR “relative energy deficiency in sport”).

The reference lists of retrieved primary studies, reviews, and relevant consensus or position statements were also manually searched to identify additional literature. Supplementary targeted searches were undertaken where required to identify mechanistic or clinical evidence relevant to the application areas examined. As this review was designed as a narrative synthesis focused on applied sport practice, studies were selected based on their relevance to BFR protocol design, physiological and performance adaptations, athlete management, and practical application.

### 2.2. Study Selection

Studies were included if they examined one or more of the following:

- Low-intensity BFR cardiovascular exercise;
- Moderate-to-high-intensity, anaerobic, sprint, or interval-based BFR conditioning;
- Sport-specific BFR conditioning, including small-sided games and mixed-modality training;
- Ischemic preconditioning (IPC) or ischemic intra-conditioning (IIC) for performance, recovery, or training adaptation;
- BFR for pain modulation, tendinopathy, rehabilitation, or maintenance of training capacity during periods of restricted loading;
- BFR-related bone metabolism, bone health, or bone stress injury management;
- Study designs including randomized controlled trials, controlled trials, crossover or within-subject designs, quasi-experimental studies, and relevant case studies;
- Studies involving trained or athletic populations participating in structured sport or exercise training and/or competitive sport.

For conditioning and performance applications, priority was given to studies involving trained individuals and competitive athletes. Where athlete-specific evidence was limited, studies involving physically active populations were considered when the intervention or outcome demonstrated clear relevance to athletic practice.

While the primary focus of this review was research conducted in trained or athletic populations, selected studies involving clinical or non-athletic populations were also considered where comparable athlete-specific evidence was limited or unavailable. This was particularly relevant to pain mechanisms, bone turnover, and bone health, where such studies provide mechanistic or clinical grounding from which potential applications to athletic populations may be cautiously extrapolated. These findings were not interpreted as direct evidence of equivalent responses in athletes.

Studies were excluded where BFR or occlusion methodology was insufficiently described, outcomes were unrelated to the application domains of this review, or the publication was not available in English. Animal and cellular studies were not included within the intervention study summaries but were considered where relevant to mechanistic interpretation.

A single reviewer (primary author) conducted the search and study selection, with consultation among all authors for ambiguous cases. We acknowledge that this selective approach may introduce selection and language biases and may favor studies with positive or applied outcomes.

### 2.3. Data Extraction and Synthesis

For each included primary study, we extracted:

- Population characteristics (training status, sport, sex, sample size);
- Study design;
- BFR or occlusion protocol details (cuff pressure, cuff width, restriction modality, and timing);
- Exercise protocol details (modality, intensity, volume, frequency, and intervention duration);
- Outcome measures (physiological, performance, pain, functional, and bone-related outcomes).

Where reported in the original publications, effect sizes were extracted to assist in the interpretation of the magnitude of responses. Standardized effect sizes were not calculated independently due to inconsistent reporting across studies and the limited availability of original data. Statistical significance was reported as presented by the original authors.

As this review was designed as a narrative synthesis, no formal quality assessment tool or pooled statistical analysis was applied. Findings were synthesized narratively and organized thematically according to low-intensity BFR cardiovascular exercise, moderate-to-high-intensity conditioning, sport-specific conditioning, IPC and IIC, joint and tendon pain management, and bone health and stress injury management.

### 2.4. Coverage of Available Literature

While this review is narrative in design, the multi-database search strategy and manual reference-list screening were intended to provide broad coverage of the literature relevant to the specific BFR applications examined. The available evidence varied considerably between domains. Athlete-specific research was more readily available for cardiovascular conditioning, sport-specific training, and performance applications, whereas direct evidence in athletic populations was considerably more limited for pain mechanisms, bone turnover, and bone health.

Accordingly, the intervention study summaries prioritize research with direct relevance to trained and athletic populations, while supporting clinical and mechanistic literature is incorporated where required to provide context and establish physiological or clinical plausibility. Where findings from non-athletic populations are discussed, these are interpreted as supportive or extrapolative evidence rather than direct evidence of efficacy in athletes.

This review was intentionally designed as a narrative rather than systematic review. Consequently, formal systematic-review procedures, including prospective protocol registration, duplicate independent screening, PRISMA reporting and flow-diagram construction, formal risk-of-bias assessment, and quantitative meta-analysis, were not undertaken. The aim was instead to provide a broad, applied synthesis of the available evidence and translate this evidence into practical considerations for strength and conditioning and sports medicine practitioners.

## 3. Low-Intensity Aerobic BFR Exercise

Low-intensity BFR-CV is typically performed at submaximal workloads (e.g., walking, cycling, rowing, or swimming) with cuffs inflated throughout the exercise bout. In athletic and well-trained populations, studies examining the addition of BFR to low-intensity aerobic exercise have established that even sub-threshold workloads can elicit cardiovascular and peripheral adaptations. For example, Park and colleagues [24] recruited collegiate basketball players to complete two weeks (twice daily, 6 days·week^−1^) of low-intensity walk training with or without BFR. The BFR group demonstrated significant increases in maximal oxygen capacity (+11.6%) and maximal minute ventilation (+10.6%), whereas no significant changes occurred in the non-BFR group. Held et al. extended this work in two endurance modalities. In international-level rowers, 5 weeks of low-intensity rowing (40–50% VO_2max_) with BFR significantly improved VO_2max_ (+9%), maximal aerobic power (+15%), and rowing time-to-exhaustion in rowers [25]. In competitive swimmers, five weeks of low-intensity swimming performed below the first lactate threshold with upper-limb BFR increased VO_2peak_ by approximately 0.3 L·min^−1^, whereas no meaningful change was observed in the non-BFR control group [26]. The swimming velocity corresponding to the first lactate threshold was largely unchanged, suggesting that the adaptations were primarily driven by improvements in maximal oxygen-transport capacity and peripheral oxidative support rather than a shift in submaximal lactate-threshold performance. Collectively, these studies confirm that low-load aerobic BFR can stimulate both central and peripheral improvements in oxygen delivery and utilization while minimizing additional musculoskeletal and mechanical training stress. Table 1 summarizes low-intensity BFR-CV interventions conducted in trained or competitive cohorts, including modality, dose (frequency and duration), and cuff prescription.

These findings are particularly relevant for strength and conditioning (S&C) coaches managing athletes during periods of high training load, during injury rehabilitation requiring mechanical load reduction, or when maintaining aerobic capacity during strength-focused mesocycles. The ability to achieve meaningful cardiovascular adaptations with minimal musculoskeletal stress positions low-intensity BFR-CV as a strategic tool within periodized training programs.

From a programming standpoint, low-intensity BFR-CV appears most suitable as a short-term intervention (e.g., 3–6 weeks) embedded within preparatory macrocycles or as a substitute for higher-intensity conditioning when mechanical loading must be reduced. Coaches should note that improvements are not uniform across all endurance markers; VO_2max_/VO_2peak_ is most consistently enhanced, whereas lactate-threshold velocity outcomes may require either longer interventions or concurrent higher-intensity work. Session design should prioritize controlled, continuous bouts at clearly defined sub-threshold intensities, with cuff pressures individually prescribed relative to arterial occlusion pressure to reduce variability in the hemodynamic stimulus.

Collectively, these data provide the evidence base underpinning the practical prescription parameters. To translate these findings into applied programming, Table 2 provides evidence-informed prescription ranges for low-intensity BFR-CV that accommodate both traditional 3–12-week blocks (2–3 sessions/week) and short “intensive” microcycles (1–2 sessions/day for 1–2 weeks) where coaches require a rapid aerobic stimulus. The prescription guidelines represent a synthesis of the available evidence alongside established BFR practice and should be interpreted as applied recommendations rather than prescriptive protocols.

## 4. Moderate-to-High-Intensity BFR Conditioning

Research examining BFR during moderate-to-high-intensity cardiovascular exercise in trained populations is limited but growing (Table 3), with most evidence derived from cycling models [29,30,31]. Two studies have evaluated short-duration repeated-sprint cycling under BFR. Giovanna and colleagues employed a two-week protocol of repeated 10-s cycling sprints and reported improvements in peak aerobic power and oxygen deficit tolerance that were comparable to performing the same protocol under systemic hypoxic conditions [29]. However, in this short, 2-week intervention, there was no improvement in aerobic capacity measures. Bourgeois and colleagues [30] extended this repeated-sprint approach to three weeks using 15-s maximal cycling sprints and observed increases in maximal aerobic power of 4.5 percent and total mechanical work of 3.6 percent, with no change in VO_2peak_. These findings suggest that brief anaerobic BFR cycling primarily enhances local peripheral qualities and anaerobic performance without inducing substantial central cardiovascular adaptation.

In contrast, Tangchaisuriya et al. (2021) implemented a 12-week high-intensity interval training (HIIT) program in trained masters road cyclists using 4-min intervals at 80 percent of peak power output [31]. Both HIIT and HIIT with BFR improved VO_2max_, stroke volume, and maximal cardiac output; however, only the BFR group demonstrated increases in total lean mass, quadriceps muscle cross-sectional area, isokinetic knee extension torque, and vastus lateralis and rectus femoris thickness. These muscular adaptations are consistent with the broader BFR literature in healthy and clinical populations, where low–moderate-intensity BFR cycling stimulates hypertrophy through amplified metabolic stress and fast-twitch fiber recruitment [32]. The BFR group also exhibited a reduction in tissue saturation index (TSI), indicating a greater degree of local deoxygenation during work intervals. Reductions in TSI indicate an imbalance between oxygen delivery and utilization within the muscle and are used as an index of intramuscular metabolic stress [33]. This desaturation response is an important mechanistic signal, as local hypoxia and metabolite accumulation during BFR are proposed to stimulate mitochondrial biogenesis, upregulate glycolytic enzyme activity, and augment motor unit recruitment [34,35,36]. The observed decline in TSI therefore provides physiological evidence that BFR meaningfully intensified the internal load of each interval, consistent with the greater hypertrophic and strength gains observed only in the BFR condition [37,38].

Researchers have applied BFR to sprint-based training [23]. Six weeks of BFR sprint training in moderately trained individuals using repeated 100 metre efforts performed at 70 percent of maximal sprint intensity improved 100 metre performance by approximately 0.4 s. While this represents a measurable change, the absolute performances remained well outside competitive standards for elite sprinters [39]. Additionally, the program consisted solely of submaximal sprint repetitions without the complementary strength development, acceleration work, or maximal velocity exposures that underpin sprint progression in trained or elite athletes. While these findings demonstrate that BFR can augment metabolic stress during submaximal sprint training, the practical application for competitive sprinters remains limited. The protocol’s lack of maximal velocity exposure, acceleration development, and integrated strength work limits its transferability to elite performance contexts. However, BFR sprint training may serve as a useful adjunct during phases requiring reduced mechanical loading (e.g., early rehabilitation, deload periods) or as a metabolic stimulus within comprehensive speed development programs. Coaches should view this application as complementary rather than foundational to sprint training.

## 5. Sport-Specific BFR Conditioning

Beyond cycling and running models, data on BFR integration into sport-specific conditioning and small-sided game formats are emerging. Such approaches to sport-specific BFR allow athletes to accumulate technical and tactical practice while simultaneously increasing metabolic stress, thereby influencing both the training stimulus and resulting adaptations. In futsal players, three weeks (10 sessions) of small-sided games performed with BFR significantly improved knee extensor and flexor strength (↑ 30.9%, ↑ 23.8%, respectively) and resulted in a 12.2% improvement in Wingate average power compared with controls [40]. In basketball players, four weeks (eight sessions) of wearing BFR during 3-versus-3 small-sided games produced significantly greater improvements in Wingate cycling performance (↑ 4.6–9.5% vs. ↑ 0.3–4.6%), countermovement jump height (↑ 6.8% vs. ↓ 0.7%), and repeated sprint ability (↓ 1.1% vs. ↑ 1.7%) relative to controls [41]. Despite higher perceived exertion, these findings indicate that BFR can meaningfully intensify basketball-specific conditioning and improve neuromuscular performance. A broader application of BFR has been demonstrated in soccer. Hosseini Kakhak and colleagues [42] applied BFR throughout multi-component training that included small-sided games, plyometrics, continuous running, and technical drills. After six weeks, the BFR group outperformed controls in muscular endurance (74.8% vs. 4.0%), change of direction ability (8.1% vs. 2.8%), and soccer-specific aerobic capacity, evidenced by larger gains in both multistage fitness test distance (54.1% vs. 24.7%) and soccer-specific (Hoff) test performance (58.4% vs. 22.7%). These findings demonstrate that BFR may amplify the metabolic and neuromuscular stimulus of sport-specific conditioning while preserving technical and tactical exposures. Continuous BFR applications, such as those used in futsal and soccer, appear to produce robust strength and endurance adaptations and, during basketball play, can enhance repeated-sprint, jump performance and anaerobic performance.

When implementing BFR during sport-specific conditioning, coaches should consider several practical factors. First, BFR sessions should be strategically placed within the weekly microcycle to avoid interference with technical training or competition. Second, athletes require familiarization with BFR sensations before applying it to complex sport-specific movements. Third, monitoring perceived exertion and movement quality is essential, as the increased metabolic stress may compromise technical execution if intensity or pressure is excessive. Finally, BFR sport-specific conditioning appears most appropriate during preparatory phases or GPP blocks or as a maintenance strategy during competitive periods when training volume is reduced. When systematically incorporated, the strategic use of BFR in team-sport conditioning offers a practical method for elevating internal load and enhancing training outcomes without increasing mechanical stress. Table 4 presents guidelines for applying BFR during moderate-to-high-intensity conditioning and sport-specific drill formats. These guidelines align conventional HIIT/repeated-sprint prescription with BFR-specific considerations and should be interpreted as applied recommendations rather than prescriptive protocols.

## 6. Ischemic Preconditioning

Ischemic preconditioning (IPC) represents a distinct application of BFR that differs fundamentally from the continuous occlusion methods described in preceding sections. Whereas BFR-CV involves sustained restriction during exercise, IPC consists of brief, intermittent cycles of limb occlusion followed by reperfusion without concurrent exercise. This passive preconditioning strategy has gained attention in sports science for two primary applications: (1) mitigation of muscle atrophy during immobilization periods and (2) augmenting subsequent athletic performance and recovery. While the protective effects of IPC during immobilization have been documented [43,44,45,46], this section focuses specifically on performance and recovery applications in trained athletes and competitive sport contexts. IPC protocols typically involve 3–4 cycles of 5-min occlusion alternating with 5-min reperfusion periods using pneumatic cuffs inflated to high pressure (≥80% AOP or ~200–220 mmHg). The ergogenic effects are attributed to vascular adaptations (enhanced nitric oxide bioavailability, improved endothelial function, increased post-reperfusion blood flow), neuromuscular modulation (altered central nervous system excitability, reduced inhibitory signals), and metabolic priming (release of vasoactive substances and growth factors) [47,48,49].

### 6.1. Acute Pre-Competition IPC

When administered immediately before competition or training (0–45 min prior), IPC has shown mixed but often positive effects on anaerobic power, sprint performance, and strength outcomes (Table 5). The magnitude of benefit is typically small (1–3%), though potentially meaningful in high-performance contexts where marginal gains determine competitive outcomes [50,51]. For example, in high-intensity repeat running sprints, acute IPC produced non-significant but directionally positive changes in maximal power (+2.6%), mean power (+0.7%) and sprint time [52]. Sprint cycling studies report varied responses. Well-trained team athletes performing 5 × 6 s maximal cycle sprints showed no improvement in peak or total power following IPC at 220 mmHg [53], whereas physically active males using a similar IPC prior to performing 12 × 6 s maximal sprints demonstrated significant improvement in mean and peak power for sprints 1–3 relative to a low-pressure placebo [11]. These contrasting findings may reflect differences in timing (5 min vs. 30 min pre-exercise), training status, or individual response variability.

In sport-specific tasks, IPC often attenuates physiological strain rather than directly enhancing performance. Elite male judo athletes performing a Special Judo Fitness Test did not show improved judo-specific performance following IPC, but experienced significantly lower heart rate and blood lactate responses compared to control, indicating reduced cardiovascular and metabolic strain during maximal efforts. IPC appears to hold greater potential in resistance exercise endurance [8,9]. In a randomized double-blind study, IPC (220 mmHg, 3 × 5 min ischemia/reperfusion, applied 4 h prior) resulted in significant increases in maximal repetitions performed at 50% 1RM for both bench press (11.3%) and 45° leg press (37.5%) [8]. Similarly, strength-trained men performing maximal effort knee extensions (3 sets × 12 reps @12RM) reported an increase in repetitions in sets 1 and 2 with IPC (200 mmHg, 3 × 5 min cycles) compared to a low-pressure placebo intervention [9]. The positive influence on performance was particularly noted in the lower limbs, suggesting that the size of the area subjected to ischemia (thigh vs. arm) may influence the magnitude of the effect.

### 6.2. IPC for Multi-Bout Performance and Recovery

While acute IPC may enhance isolated maximal efforts, many sporting contexts require repeated high-intensity efforts over the course of a day or tournament format. Research has examined repeated or intra-session IPC applications to investigate the ergogenic response and recovery across successive efforts. Two primary strategies have been identified: (i) prior to and between repeated maximal efforts and (ii) ischemic intra-conditioning (IIC), where brief occlusion is applied during intra-session rest intervals to influence performance decay and recovery kinetics (Table 6).

#### 6.2.1. Repeat-Bout Efforts in Competition Format

In competitive environments requiring repeated bouts of high-intensity efforts separated by short recovery windows, IPC has demonstrated utility in maintaining performance capacity and enhancing recovery kinetics. To illustrate this, well-trained male kayak athletes completed two 1000-m time trials (TT1 and TT2) separated by 80 min under three experimental conditions: control (CON), IPC before TT1 only (IPC1), and IPC before TT1 and TT2 (IPC2). When IPC was administered prior to TT1 (IPC1 and IPC2 conditions), athletes demonstrated a significant improvement in performance relative to control, completing the first time trial 6.9–8.5 s faster (ES = −1.24 to −1.53, *p* < 0.01), with corresponding increases in average power (19–23.8 watts; ES = 0.89–1.12; *p* < 0.05). However, in the IPC1 condition, this ergogenic effect did not carry over to TT2, with no mean difference in 1000 m time or power output compared to control. In contrast, applying IPC before both efforts (IPC2) produced the most robust and sustained improvements. This performance advantage was retained for TT2, reducing 1000-m completion time by 6.3 s relative to control (ES: −1.26, *p* < 0.05) and increasing average power by 17.1 watts (ES = 1.18, *p* < 0.05). These findings suggest that while IPC applied prior to a single maximal bout can transiently enhance performance, the greatest and most sustained ergogenic effect occurs when IPC is repeated before each maximal effort during multi-bout competition formats.

#### 6.2.2. Post-Exercise Recovery

IPC has shown potential as a post-exercise recovery modality, with some studies reporting both immediate and delayed benefits. When applied after strenuous lower-body exercise, IPC (220 vs. 15 mmHg; 2 × 3 min ischemia/reperfusion) improved immediate and subsequent performance 24 h later [60]. Immediately post-intervention, IPC produced a moderately beneficial effect on squat jump height (ES = 0.63). At 24 h post-exercise, IPC produced small-to-large beneficial effects across multiple jump-derived variables, including improved concentric (ES = 0.36) and eccentric (ES = 0.26) peak velocity in countermovement jump, and large improvements in squat jump eccentric power (ES = 1.38) and eccentric acceleration (ES = 1.24). Sprint recovery was also enhanced, with cumulative 10- and 40-m sprint times recovering more rapidly in the IPC condition.

In elite judo athletes, although IPC (3 × 5 min ischemia/reperfusion) did not influence Special Judo Fitness Test outputs, it produced recovery advantages post-exercise [61]. Compared with placebo (20 mmHg), IPC resulted in significantly improved heart rate recovery (14–18 beats per minute lower), 11.6% greater reduction in blood lactate, higher CMJ performance and reduced muscle soreness (−0.8 points) at 60 min post-test. In endurance contexts, IPC applied during short recovery windows (45 min) between repeated 5 km cycling time trials was equally effective as active recovery or neuromuscular electrical stimulation (NMES) in maintaining performance and restoring muscle blood volume [59]. These findings suggest that IPC may support neuromuscular and physiological readiness during periods of dense training or competition, although the available evidence remains limited and context-dependent.

Effective IPC implementation depends on the competitive format, recovery window, and the athlete’s tolerance to high-pressure occlusion. Table 7 provides applied guidelines for selecting IPC timing and cycle structure to support pre-event priming and between-bout recovery without disrupting standard preparation and should be interpreted as applied recommendations rather than prescriptive protocols.

#### 6.2.3. Ischemic Intra-Conditioning

Beyond pre-competition priming and between-bout recovery, the occlusion–reperfusion stimulus can also be embedded within the training session itself as ischemic intra-conditioning (IIC). In this approach, an occlusion bout is applied during inter-repetition rest intervals and released before the subsequent work effort, with the intent of leveraging IPC-like vascular and metabolic signaling while preserving the external demands and technical quality of the session. Conceptually, IIC is designed to leverage the systemic and local protective and potentiating effects associated with transient ischemia during the high-metabolic-stress periods created between repeated efforts. The proposed objectives are twofold: first, to mitigate fatigue-induced performance decline across repeated efforts and, second, to potentiate molecular signaling pathways that contribute to physiological adaptations.

In acute resistance and sprint training studies, the effects of IIC appear to be minimal. Across six 30-m maximal sprints performed with 7-min recovery, well-trained athletes showed no improvement in sprint times with IIC (5 min at 60% or 80% AOP) compared with control [55]. In resistance-trained men performing bench press (8 sets × 2 repetitions at 20–90% 1RM), IIC applied before the first set and during subsequent inter-set rest intervals resulted in meaningful increases in peak bar velocity at light-to-moderate loads (20% and 50% 1RM) with small-to-moderate effects [57].

When examined over longer periods (2–4 weeks), IIC appears to stimulate meaningful physiological adaptation [14,62]. Two studies using the same protocol investigated 4 weeks (twice weekly) of cycling sprint-interval training (SIT) with IIC applied during each recovery interval. VO_2max_ increased significantly (4.5–5.9%), whereas control groups reported no change. Despite these physiological improvements, neither study observed significant changes in time-trial performance, suggesting that improvements in maximal aerobic capacity did not translate to enhanced endurance performance within the relatively short training duration.

Both studies also identified favorable shifts in molecular and cellular markers associated with hypoxic signaling and physiological adaptations. In the IIC training groups only, increases were observed in hypoxia-inducible factor-1α (HIF-1α), implicating activation of angiogenic pathways, and Ki-67, indicating increased cellular proliferation associated with capillary expansion and muscle remodeling [62,63]. Collectively, these findings suggest that IIC during high-intensity training may provide an additional vascular and cellular signaling stimulus that contributes to the observed improvements in aerobic capacity.

Table 8 provides guidelines for integrating IIC within SIT/HIIT microcycles, emphasizing pressure selection, timing of inflation relative to work bouts, and occlusion duration that preserves interval quality. These recommendations align IIC-specific constraints with established interval-training principles while highlighting its primary role as a chronic signaling stimulus rather than an acute ergogenic strategy. The following guidelines represent a synthesis of the available evidence alongside established BFR practice and should be interpreted as applied recommendations rather than prescriptive protocols.

### 6.3. Summary and Recommendations

Ischemic preconditioning represents a distinct and potentially valuable application of blood flow restriction technology in athletic settings, though its effects are more nuanced than continuous BFR methods. For pre-performance use, IPC can be applied immediately before the standard warm-up, typically leaving approximately 30–45 min before short-duration, high-intensity efforts (30–90 s). IPC may also be used as a recovery strategy between bouts in tournament and multi-day competition formats. Performance improvements, when present, are typically small (1–3%) but may be meaningful in high-performance contexts where marginal gains are competitively significant.

However, several important limitations warrant consideration. Individual response is variable, with clear responders and non-responders across studies. Protocol parameters (cuff pressure, cycle duration, timing) vary widely in the literature, making specific recommendations challenging. The mechanisms underlying IPC’s ergogenic effects remain incompletely understood, and the relationship between acute performance enhancement and chronic training adaptations requires further investigation. Additionally, most research involves laboratory-based performance measures; evidence for sport-specific performance improvements in competitive settings is limited.

For S&C coaches, IPC should be viewed as a supplementary, context-specific tool rather than a primary training method. Successful implementation requires systematic individual testing during training periods to identify responders, careful protocol standardization, and realistic expectations regarding effect magnitude. Even in the absence of objective performance gains, athletes who perceive benefit from IPC may experience meaningful placebo or confidence effects that support its continued use, particularly in sports where psychological readiness is critical [64,65,66].

When applied judiciously in appropriate contexts with proper athlete selection and monitoring, IPC offers an evidence-informed strategy for pre-competition priming and between-bout recovery that complements the broader BFR toolkit outlined in this three-part series. However, coaches must balance the potential small performance gains against the time, equipment, and monitoring requirements, ensuring that IPC implementation does not compromise the fundamental aspects of training and competition preparation. Specific protocols for acute/between-bout IPC applications and ischemic intra-conditioning for chronic adaptations are summarized in Table 7 and Table 8, respectively.

## 7. BFR for Joint and Tendon Pain Management

Joint and tendon pain represent a significant challenge in athletic populations, often limiting training participation and reducing performance [67,68]. Traditional management varies depending on the injury; however, during critical training or competition periods, there is often a requirement to modify physical activity to manage symptoms, which can compromise preparation and performance [69,70].

BFR training has emerged as a potential solution, offering a method to attenuate pain whilst maintaining or improving muscle strength and function [6,71,72,73,74,75,76]. This is relevant for athletes with tendon or joint issues who experience clinically meaningful pain during exercise. In these cases, training intensity is often reduced to avoid symptom exacerbation, sometimes below the threshold required to elicit meaningful physiological adaptation [77]. The analgesic effects of BFR exercise appear to arise from multiple complementary mechanisms, including exercise-induced hypoalgesia via activation of endogenous opioid pathways and endorphin release [78,79], as well as potential anti-inflammatory effects through modulation of pro-inflammatory cytokines [80,81]. These mechanisms position BFR as a practical tool for managing pain during training while simultaneously addressing strength and muscle mass deficits that often accompany training load reduction [82,83].

Single-session studies demonstrate immediate analgesic effects of BFR exercise [6,71,84]. In individuals with anterior knee pain, one bout of low-load (4 sets @ 30% 1RM) knee extension exercise with BFR (80% AOP) significantly improved functional movements and reduced pain during and post-exercise, with effects sustained for at least 45 min [6]. In a randomized controlled trial of adults with patellofemoral pain, four weeks of low-load (30% 1RM) hip and knee strengthening with BFR produced reductions in pain and improvements in function comparable to conventional high-load training (70% 1RM), with significantly greater reductions in worst pain post-treatment and greater gains in knee extensor strength at follow-up in the BFR group [85].

Longitudinal studies demonstrate that low-load (30% 1RM) BFR training can produce meaningful improvements in pain, strength, and function across chronic musculoskeletal conditions while using substantially lower external loads than traditional resistance training [74,76,86]. In individuals with patellofemoral pain, eight weeks of quadriceps strengthening at 30% 1RM with BFR (3 sessions·wk^−1^) produced improvements in knee-related function scores and worst pain comparable to traditional training at 70% 1RM while yielding significantly greater reductions (*p* ≤ 0.02) in pain during activities of daily living and greater recovery of knee extensor strength [76].

In male collegiate athletes with chronic non-specific low back pain, four weeks of upper and lower body BFR resistance training at 30% 1RM significantly reduced pain intensity and disability (*p* < 0.001) and increased trunk flexor and extensor endurance, with no between-group differences relative to high-load training at 70% 1RM [86]. In an applied sport context, a collegiate decathlete with patellar tendinopathy completed a ten-week in-season lower-body BFR resistance training program (30% 1RM; 2 sessions·wk^−1^). The addition of the BFR intervention was associated with improvements in pain, self-reported knee function, lower-limb strength, and tendon structure, allowing the athlete to continue training and competition without symptom exacerbation [74]. Table 9 summarizes guidelines for integrating BFR as a pain-modulation strategy within athlete management and should be interpreted as applied recommendations rather than prescriptive protocols.

## 8. BFR for Bone Health and Stress Injury Management

Bone stress injuries represent a significant concern in athletic populations, particularly among endurance athletes and those in weight-sensitive sports, with injury burden amplified by repetitive loading demands and, in some cohorts, low energy availability and hormonal disruption [87,88,89]. Traditional management commonly requires a period of substantial load modification and staged return to activity, with return-to-sport timeframes ranging from several weeks to months depending on severity and anatomical site [88,89]. Beyond bone stress injuries, long-term bone health is a parallel concern in athletes with low bone mineral density (BMD), particularly those with menstrual dysfunction and/or Relative Energy Deficiency in Sport (RED-S), where low energy availability can impair endocrine function and subsequent bone health [87]. In these contexts, the applied challenge for S&C coaches is maintaining muscle mass and fitness while conventional osteogenic loading strategies are restricted.

The mechanisms by which BFR exercise might influence bone metabolism differ from traditional mechanical loading models, where peak strain magnitude and strain rate are key determinants of the osteogenic response [90]. In contrast, BFR applied during low-load exercise creates a unique physiological environment that may elicit bone adaptations through indirect signaling pathways. Evidence suggests two complementary mechanistic pathways may contribute to BFR-mediated bone adaptation: (1) muscle-to-bone endocrine signaling through myokine production and myogenic factors and (2) hypoxia-induced activation of transcriptional pathways regulating angiogenesis and osteogenesis [1,91].

During BFR training, metabolic stress stimulates skeletal muscle to produce myokines and growth factors, which regulate osteoblast and osteoclast function [92,93]. Additionally, venous outflow restriction has been proposed to alter the intraosseous fluid environment, with experimental work demonstrating that changes in intramedullary pressure can induce intracortical fluid flow and influence bone mechanotransduction pathways and osteocyte signaling [94,95]. A secondary complementary pathway is hypoxia-sensitive signaling, particularly activation of hypoxia-inducible factor-1α (HIF-1α), a master transcriptional regulator that increases vascular endothelial growth factor (VEGF) expression in osteoblasts, coupling angiogenesis to osteogenic differentiation during skeletal development and repair [96]. Although direct evidence that BFR activates these pathways within bone tissue in athletic populations is limited, BFR exercise has been reported to induce hypoxia-response transcripts in non-athletic populations, supporting mechanistic plausibility for a pro-angiogenic and pro-remodeling milieu when mechanical loading is constrained [97,98,99].

Bone turnover markers reflect dynamic remodeling processes and include formation markers (e.g., P1NP, bone alkaline phosphatase) and resorption markers (e.g., CTX) [100]. In older men, six weeks of low-intensity resistance training with vascular restriction increased bone alkaline phosphatase by approximately 21% and improved the bone alkaline phosphatase-to-CTX ratio, suggesting a shift favoring bone formation [101]. In postmenopausal women with osteoporosis or osteopenia, 12 weeks of low-intensity resistance training with BFR improved strength and increased P1NP [102].

Evidence for BMD change from BFR is limited and often constrained by study duration, with many interventions shorter than the time required for robust skeletal adaptation [100]. In applied clinical scenarios, the most defensible pattern is attenuation of bone loss rather than clear osteogenic gains. Accordingly, BFR should not be positioned as a substitute for impact or heavy resistance training where those are appropriate and tolerated [93,103]. Instead, BFR is best framed as a supplementary strategy when osteogenic loading is temporarily contraindicated [104]. In practice, this may include integrating low-load BFR resistance training during early rehabilitation phases, employing contralateral strength training when one limb is load-restricted, and using passive BFR protocols to attenuate disuse-related muscle loss during immobilization or severe unloading, before progressing toward conventional loading as clearance permits.

Key limitations warrant explicit acknowledgement: athlete-specific trials remain scarce, and bone-oriented protocols (pressure, load, frequency, and duration) are not established [1,105]. As such, BFR should be implemented within a multidisciplinary plan, with realistic expectations that long-term bone health improvements require restoration of energy availability and endocrine function alongside progressive reintroduction of osteogenic loading. Accordingly, implementation should prioritize interdisciplinary clearance, conservative prescription, and monitoring. Table 10 summarizes evidence-informed integration guidelines for strength and conditioning practitioners working with athletes affected by bone stress injury, low BMD, or RED-S. The following guidelines represent a synthesis of the available evidence alongside established BFR practice and should be interpreted as applied recommendations rather than prescriptive protocols.

## 9. Conclusions

Evidence suggests that BFR extends beyond resistance training to support cardiovascular conditioning, pre-performance priming, and selected clinical-management applications. Low-intensity BFR cardiovascular exercise provides a meaningful aerobic stimulus with minimal mechanical stress, which may be valuable during rehabilitation, deload phases, and periods of high running or impact exposure. Moderate- to high-intensity and sport-specific BFR applications may augment internal load during interval-based work and small-sided game formats, improving aerobic power, repeated-sprint qualities, and selected neuromuscular outcomes while preserving technical and tactical specificity. Ischemic preconditioning represents a related occlusion–reperfusion strategy that may produce small, context-dependent improvements in short-duration, high-intensity performance; however, variability in responsiveness supports individual trialing prior to competition use. Emerging therapeutic applications further position BFR as a versatile adjunct when pain or tissue tolerance constrains conventional loading. Across acute and longitudinal evidence, BFR can attenuate pain while maintaining or improving strength and functional outcomes in selected joint and tendon presentations, provided implementation remains within scope of practice and is coordinated with medical staff. In bone stress and low-BMD contexts, BFR should be framed as a bridge strategy to preserve muscle and conditioning when osteogenic loading is temporarily contraindicated, rather than as a primary method for increasing BMD.

S&C coaches should therefore treat BFR as a supplementary modality that complements, rather than replaces, established training and rehabilitation principles. Best practice requires individualized pressure prescription, conservative progression, appropriate integration within periodized plans, and systematic monitoring of symptoms, movement quality, and recovery. Key limitations include heterogeneous protocols, limited athlete-specific evidence across several domains, and uncertainty regarding long-term outcomes and optimal dose–response relationships. Future research should prioritize longer interventions, sport-specific endpoints, female and youth cohorts, and clinical trials in bone stress and RED-S populations. Together with Parts 1 and 2, this series provides recommendations based upon the current reviewed evidence for integrating BFR across performance, load management, and return-to-training contexts to support athlete health and performance. S&C coaches should therefore interpret the available evidence in the context of the individual athlete, training objectives, clinical considerations, and competitive environment when determining whether and how BFR is integrated into practice.

## Figures and Tables

**Table 1:** Low-intensity BFR-CV Training Studies in Athletic Populations.

Study/Design	Population	Cuff Pressure/Width	Protocol	Key Findings	Applied Insights
Park et al. [24] *Randomized 2 groups*	Male college basketball players; BFR *n* = 7; CON *n* = 5	160–230 mmHg; cuff width NR; C-BFR (maintained during rest)	Exercise: walking: 5 × 3-min at 4 km·h^−1^, 5% grade; 1-min rest Dose: 2 wk; 2×/day; 6 d·wk^−1^	↑ VO_2max_ 11.6% ^1,2^ (CON −1%) ↑ VE_max_ 10.6% ^1^ ↑ Wingate anaerobic capacity 2.5% ^1^ ↑ SV 21.4%	Implement during rehabilitation, during deload weeks, or when managing high training loads.
Held et al. [25] *Randomized 2 groups*	Elite rowers; BFR *n* = 16; CON *n* = 15	Elastic bands at 75% of max stretch; 13 cm cuff width; C-BFR	Exercise: rowing: 2 × 10 min at [La−]_b_ < 2 mmol·L^−1^; 10 min rest Dose: 5 wk, 3 d·wk^−1^	↑ VO_2max_ (9.1% v CON 2.5% ^^^) ^1,^^^ ↑ power at VO_2max_ 15.3% ^1^	BFR can improve the physiological response to low-intensity rowing; useful when intensity must stay low (fatigue management/return-to-training) but adaptation is still targeted.
Held et al. [26] *Randomized 2 groups*	Competitive male swimmers; BFR *n* = 10; CON *n* = 8	75% AOP; 8 cm cuff width; C-BFR	Exercise: swimming 2 × 10 min; 10 min rest. Dose: 5 wk, 3 d·wk^−1^ Test: 7 × 200 m IST	↑ VO_2peak_ 0.29 L·min^−1^	Low-intensity BFR swimming can improve aerobic capacity; should complement threshold training for race-pace development.
Chen et al. [27] *Randomized 2 groups*	Endurance-trained males; BFR *n* = 10; CON *n* = 10	1.3 × SBP; 14.2 cm cuff width; C-BFR	Exercise: treadmill running: 5 × 3-min at 50% HRR; 1-min rest Dose: 8 wk, 3 d·wk^−1^	↑ VO_2max_ 5.1% ^1^ (CON −1.1%) ↑ Isokinetic knee extension 16.5% ^1,2^ (CON −5.9%) ↑ Thigh muscle mass ↔ T:C Ratio BFR, ↓ No-BFR	BFR running enhances aerobic function, muscle mass, and quadriceps strength while maintaining anabolic balance in endurance athletes.
Thompson et al. [28] *Quasi pre–post*	Elite rowers; *n* = 31 recruited; paired analyses *n* = 20–25 depending on outcome (M = 8, F = 12–17)	80% AOP; 10 cm cuff width	Exercise: rowing 2 × 10 min at [La−]_b_ < 2 mmol·L^−1^; 10 min rest; Dose: 4 wk, 3 d·wk^−1^ (*n* = 4 trained on cycle ergometer) Test: 2000-m TT	↓ 2000-m TT: females 1.09 ± 1.2% ^1^; males 1.17 ± 0.48% ^1^ ↑ VO_2max_ females 3.0 ± 3.9% ^1^	Supports results of Held et al. [25]

Abbreviations: BFR, blood flow restriction; M, males; F, females; wk, week; d·wk^−1^, days per week; CON, control group; NR, not reported; C-BFR, continuous blood flow restriction; AOP, arterial occlusion pressure; [La−]_b_, blood lactate; SBP, systolic blood pressure; m, meters; min, minutes; HRR, heart rate reserve; T:C, testosterone-to-cortisol ratio; SV, stroke volume; TT, time trial; VO_2max_, maximal oxygen uptake; VE_max_, maximal minute ventilation; IST, incremental step test; ^^^, small effect size, ^^^^, large effect size; ↑, increase; ↓, decrease; ↔, no-change; ^1^, *p* < 0.05 compared to pre-training values; ^2^, *p* < 0.05 compared to control.

**Table 2:** Guidelines for Low-Intensity BFR-CV Exercise: Program Variables.

Variable	Guidelines	Applied Insights
Frequency	2–3 ×/week for 3–12 weeks 1–2 ×/day for 1–2 weeks (intensive blocks)	High-frequency BFR-CV without excessive fatigue due to low mechanical load.
Intensity	<45–50% VO_2max_ <45–50% HRR	Low intensity but high metabolic load due to occlusion.
Exercise Type	Walking, Cycling, Rowing, Elliptical, Ski-erg	Select modalities aligned to sport demands; prioritize those permitting stable pacing and consistent technique.
Inflation Pressure	40–60% AOP	Lower pressures are preferred due to longer continuous occlusion times.
Pressure Modality	Continuous or intermittent	Continuous preferred to maximize metabolic stress.
Restriction Time	Upper Body: ≤15 min Lower Body: ≤20 min	Multimodal improvements seen with ≥15 min occlusion duration. Supervise and monitor longer training durations or prescribe a brief deflation/inflation mid-way through the session.

Abbreviations: BFR-CV, cardiovascular blood flow restriction; %, percentage; AOP, arterial occlusion pressure; HRR, heart rate reserve; VO_2max_, maximal oxygen uptake; min, minutes.

**Table 3:** Moderate-to-High BFR-CV Training Studies in Athletic Populations.

Study/Design	Population	Cuff Pressure/Width	Protocol	Key Findings	Applied Insights
Behringer et al. [23] *Randomized 2 groups*	Trained males; BFR *n* = 12; CON *n* = 12	Elastic bands; 13 cm cuff C-BFR (maintained during rest)	Exercise: sprint running 6 × 100 m at 60–70% of MSS; 1-min inter-rep recovery; Dose: 6 wk; 2 d·wk^−1^.	↓ 100-m time −0.38 s ^2^ (CON −0.16 s) ↑ RFD ^2^ ↑ BF/RF muscle thickness ^2^	BFR can enhance adaptation to moderate-intensity sprint training; applicability to elite sprint performance remains limited without concurrent sprint and strength/power development.
Giovanna et al. [29] *Randomized 4 groups*	Endurance-trained males; BFR *n* = 10; HTG *n* = 10; BFR-REC *n* = 10; CON *n* = 9	45% AOP; 11 cm cuff width; BFR: condition specific	Exercise: HIIT Cycling 5 × (10-s max efforts + 20-s at 75 W); 4 sets; 5–10 min between set recovery Dose: 2 wk; 3 d·wk^−1^	↑ Peak aerobic power across all groups (2.2–4.4%) ↑ Maximal accumulated O_2_ deficit ↑ Accumulated VO_2_ during training	Longer blocks and/or higher exposure may be required to detect any incremental benefit.
Bourgeois et al. [30] *Randomized 2 groups*	Endurance-trained males; BFR *n* = 9; CON *n* = 8	50–70% AOP, progressed weekly; 9 cm cuff width	Exercise: HIIT Cycling: 4 × (15 s at 100% MAP + 15 s at 60 W); 6–8 sets; Dose: 3 wk; 3 d·wk^−1^	↑ Wingate total mechanical work by 3.6% ^1^ ↑ MAP by 4.5% ^1^ ↔ VO_2peak_ ↓ 5-km TT 0.8% ^1^ (CON 1.2% ^1^) ↓ TRIMP by 31% vs. CON	Short HIIT + BFR blocks can improve maximal aerobic power and sprint-work capacity while reducing internal training load (TRIMP).
Tangchaisuriya et al. [31] *Randomized 3 groups*	Trained male cyclists; BFR-IT *n* = 17; HIIT *n* = 17; CON *n* = 16	30% AOP; 11 cm cuff width	Exercise: HIIT Cycling 4 × 4 min at 80% PPO. BFR-IT: reps 1 and 3 at 80% PPO, rep 2 and 4 at 60% PPO CON: 75 min at 65–70% PPO Dose: 12 wk; 2 d·wk^−1^	↑ VO_2max_ BFR-IT 7.7% ^1,2^; HIIT 8.1% ^1^ ↑ Peak Power: BFR-IT 6.8% ^1,2^; HIIT 5.0% ^1,2^ ↑ SV and CO: BFR-IT ^1^; HIIT ^1^; CON ^1^ ↑ Maximal a–v O_2_ difference: BFR-IT 4.3% ^1^ ↓ 40-km TT: BFR-IT ^1,2^ > HIIT ^1,2^ > CON ^1^ BFR-IT: ↑ lean mass 1.2% ^1^; leg lean mass 2.1% ^1^, RF CSA 4.2%, VL CSA 8.4%	Low-pressure BFR used with HIIT cycling may amplify peripheral adaptations (a–v O_2_ difference), muscle hypertrophy and oxygen extraction.

Abbreviations: BFR, blood flow restriction; wk, week; d·wk^−1^, days per week; CON, control group; AOP, arterial occlusion pressure; a–v, arterial–venous; BF, biceps femoris; BFR-IT, blood flow restriction interval training; BFR-REC, blood flow restriction applied during recovery; C-BFR, continuous blood flow restriction (maintained during inter-repetition rest); CO, cardiac output; CSA, cross-sectional area; HIIT, high-intensity interval training; HTG, hypoxic training group; MAP, maximal aerobic power; MSS, maximal sprint speed; O_2_, oxygen; PPO, peak power output; reps, repetitions; RF, rectus femoris; RFD, rate of force development; SV, stroke volume; TRIMP, training impulse; TT, time trial; VL, vastus lateralis; VO_2_, oxygen uptake; VO_2max_, maximal oxygen uptake; VO_2peak_, peak oxygen uptake; W, watts; ↑, increase; ↓, decrease; ↔, no change; > greater than; ^1^, *p* < 0.05 compared to pre-training values; ^2^, *p* < 0.05 compared to control.

**Table 4:** Guidelines for Moderate-to-High-Intensity BFR-CV Exercise and Sports-Specific Training.

Variable	HIIT, Repeated Sprints	Sport-Specific	Applied Insights
Frequency	2–3×/week	2–3×/week for 3–6 weeks	Maximum frequency of 3×/week; longer blocks tend to yield more robust adaptations
Intensity	≥60% VO_2max_; intervals at 100% MAP (cycling) or MAS (running); sprint reps ~70% MSS	Aligned with sport demands of each drill	Adjust cuff pressure and modulate intensity to complete required task effectively under BFR
Exercise Type	Running, cycling, swimming, rowing intervals; repeated sprints	SSG; COD drills; mixed-modality conditioning	Does not replace technical or tactical training; use strategically to support performance-relevant outcomes
Inflation Pressure	30–45% AOP	Moderate to high pressures, including fixed values (e.g., 110%SBP)	Higher inflation pressures increase metabolic stress but must remain tolerable; longer intervals may require lower pressures
Pressure Modality	Continuous or intermittent	Primarily continuous	Intermittent inflation can reduce fatigue and preserve mechanics; continuous is feasible for SSG/multi-component work
Session RPE Monitoring	RPE reporting of 1–2 points higher than non-BFR is common		Essential for managing fatigue and adjusting inflation pressure and session intensity
Technical Quality	Deflate cuffs if technique or physical performance output degrades > 10% from baseline		Maintain sport-specific movement quality. BFR should challenge conditioning without compromising skill execution
Restriction Time	Upper Body: ≤15 min; Lower Body: ≤20 min; Per block/bout		Adhere to safe maximum inflation times. For longer sessions, include planned reperfusion blocks before re-inflation

Abbreviations: BFR-CV, blood flow restriction cardiovascular; wk, week; %, percentage; VO_2max_, maximal oxygen consumption; MAP, maximal aerobic power; MAS, maximal aerobic speed; MSS, maximal sprint speed; HIIT, high-intensity interval training; SSG, small-sided game; COD, change of direction; AOP, arterial occlusion pressure; SBP, systolic blood pressure; CV, cardiovascular; min, minutes; BFR, blood flow restriction; RPE, rate of perceived exertion.

**Table 5:** Acute Pre-Competition Intervention Studies on Ischemic Preconditioning (IPC) and Sports Performance.

Study/Design	Population	Cuff Pressure/Width	Protocol	Key Findings	Applied Insights
Paradis-Deschênes et al. [47] *Randomized crossover*	Strength-trained men; *n* = 10	200 mmHg; sham 20 mmHg; 21 cm cuff	IPC: 3 × 5 min Test: Knee extension (5 × 5 reps) Timing: 20 min post-IPC	↑ Peak force 11.8% ^#^ ↑ Average force 12.6% ^#^ ↑ Resting muscle blood volume ~46.5% ^##^ ↑ muscle O_2_ uptake 15.8% ^#^	IPC may acutely potentiate strength output, plausibly via improved local perfusion and metabolic utilization.
Patterson et al. [11] *Randomized crossover*	Active males; *n* = 14	220 mmHg; sham 20 mmHg; 14.5 cm cuff width	IPC: 4 × 5 min Test: cycle sprints (12 × 6 s) Timing: 30 min post-IPC	↑ Peak power in sprints 1–3 (2.4%, 2.6%, 3.7%) ^#^ ↑ Mean power in sprints 1–3 (2.8%, 2.6%, 3.4%) ^#^	IPC performed pre-competition can enhance early repeated-sprint power outputs.
Gibson et al. [53] *Randomized crossover*	Well-trained team-sport athletes; *n* = 16 (F = 9, M = 7)	220 mmHg; sham 50 mmHg; cuff width NR	IPC: 3 × 5 min Test: cycle sprints (5 × 6 s) Timing: 5 min post-IPC	↔ Peak and total power; ↓ Blood lactate in females: 1.6 mmol·L^−1^ ^##^	Effects may be sex-specific and/or task-dependent; cycling testing may limit transfer for running-dominant athletes.
Dantas et al. [52] *Randomized crossover*	Trained males; *n* = 21	220 mmHg; 100% APP; sham 20 mmHg; CON; cuff width NR	IPC: 3 × 5 min Test: RAST (6 × 35 m; 10 s rest) Timing: immediately prior	↑ P_max_ by 2.6% and P_mean_ by 0.7% at 100% APP; neither statistically significant	Full arterial occlusion may induce small acute improvements; effects remain trivial and should be interpreted cautiously.
Marocolo et al. [9] *Randomized crossover*	Active males; *n* = 13;	220 mmHg; Sham 20 mmHg; 13 cm cuff	IPC: 4 × 5 min Test: Knee extension (3 × AMRAP at 12RM) Timing: 8 min post-IPC	↑ Repetitions in sets 1–2 with IPC and sham vs. control ↔ Blood lactate and fatigue index	Similar improvements in IPC and sham suggests expectancy effects may contribute, so implement only if an athlete shows repeatable individual benefit.
Santana et al. [8] *Randomized crossover*	Resistance-trained; *n* = 24; ULG: *n* = 12 (F = 4, M = 8) LLG: *n* = 12 (F = 2, M = 10)	220 mmHg; sham 40 mmHg; cuff width NR	IPC: 3 × 5 min Test: Bench press and leg press (1 × AMRAP at 50% 1RM) Timing: 4 h post-IPC	ULG: ↑ reps 11.3% *^,#^ LLG: ↑ reps 37.5% *^,##^ ↔ Sham condition	IPC can enhance strength endurance, with larger effects reported in lower limb tasks.
Ceylan et al. [54] *Randomized crossover*	Elite male judo athletes; *n* = 10	220 mmHg; CON; cuff width NR	IPC: 3 × 5 min Test: SJFT Timing: immediately prior	↔ SJFT performance ↓ HR * and blood lactate * vs. control	IPC may reduce physiological strain despite unchanged SJFT performance. May be useful for recovery management in tournament-style competition formats.

Abbreviations: F, female; M, male; ULG, upper limb group; LLG, lower limb group; IPC, ischemic preconditioning; AMRAP, as many repetitions as possible; RM, repetitions maximum; APP, auscultatory pulse pressure; CON, control; P_max_, maximum power; P_mean_, mean power; reps, repetitions; RAST, running anaerobic sprint test; SJFT, special judo fitness test; NR, not reported; HR, heart rate; ↑, increase; ↓, decrease; ↔, no change; ^#^, small effect size vs. control; ^##^, moderate effect size vs. control; *, *p* < 0.05 vs. control.

**Table 6:** Repeated-Bout, Intra-Session, and Post-Exercise Ischemic Preconditioning (IPC) in Sport and Exercise.

Study/Design	Population	Cuff Pressure/Width	Protocol	Key Findings	Applied Insights
Fostiak et al. [55] *Randomized crossover*	Well trained athletes; *n* = 34 (F = 22, M = 12)	60% AOP, 80% AOP, CON; 10 cm cuff width	IPC: 1 × 5 min. Test: 6 × 30 m sprints; 7 min inter-rep recovery. Timing: pre-test and during inter-rep rest interval	No between-group differences in sprint performance	Lower-pressure IPC did not enhance sprint performance; higher inflation pressures may be required.
Pugh et al. [56] *Randomized crossover*	Competitive road cyclists; *n* = 11	200 mmHg; 12 cm cuff	IPC: 1 × 2 min. Test: 6 × 30-s cycling at ~85% Pmax; 4.5 min inter-rep recovery Timing: 2 min during inter-rep rest interval	During exercise: ↑ VCO_2_ *^,^^ and VE *^,^^ Early Recovery (0–2 min): ↓ TSI *, VO_2_ *, VCO_2_ *, and VE * ↑ VEGF at 3 h post-exercise *^,^^^	Intra-session IPC may augment vascular/angiogenic signaling without extending work duration.
Jarosz et al. [57] *Randomized crossover*	Resistance-trained men; *n* = 10	80% AOP; 10 cm cuff width (upper limb)	IPC: 1 × 2.5 min. Test: Bench Press 8 × 2 reps (20–90% 1RM); 3 min inter-set rest. Timing: pre-set and during inter-set rest interval.	↑ peak velocity at 20% * and 50% 1RM * Small-to-moderate effects at heavier loads (60–90% RM)	Brief IPC may augment bar velocity at light–moderate loads; practical relevance likely individual-dependent.
Halley et al. [58] *Randomized crossover*	Competitive male kayakers; *n* = 8	220 mmHg; 18 cm cuff width	IPC: 4 × 5 min. Test: 2 × 1000 m kayak TT (TT1 & TT2); 80 min between trials. IPC1 = IPC before TT1 only; IPC2 = IPC before TT1 and TT2. Timing: 40 min pre-TT.	TT1: ↓ time 2.8–3.5% *^,###^, ↑ power 7–10% *^,###^ with IPC1 and IPC2 TT2: ↓ time 2.5% *^,###^, ↑ power 9.6% *^,###^ with IPC2	Repeated IPC may re-prime performance between maximal efforts in multi-bout competition.
Paradis-Deschênes et al. [59] *Randomized crossover*	Endurance male athletes; *n* = 9; (IPC, AR, NMES)	220 mmHg; 21 cm cuff width	IPC: 3 × 5 min. Test: 2 × 5 km cycling TT; 45 min inter-rep recovery. Timing: during the inter-trial recovery.	Similar TT decrement across conditions ↑ blood volume pre- TT2 13.3%	IPC appears comparable to active recovery and NMES; may be used as a complementary recovery modality.
Beaven et al. [60] *Randomized crossover*	Trained males (*n* = 10) & females (*n* = 4)	220 mmHg; sham 15 mmHg; cuff width NR	IPC: 2 × 3 min. Test: CMJ, Squat Jump, Leg Press, 6 × 40 m sprints; test repeated immediately and 24 h. Timing: applied immediately after initial test.	Immediate: ↑ Squat jump height ^##^; 24 h post: ↑ Recovery squat jump maximal power ^#^; ↑ eccentric peak power ^###^ & acceleration ^###^; ↑ CMJ concentric & eccentric velocity ^#^; ↑ Recovery cumulative 10-m & 40-m sprint times	Post-exercise IPC may improve neuromuscular recovery (immediate to 24 h) during congested training or competition schedules.
Ceylan et al. [61] *Randomized crossover*	Elite male judokas (*n* = 13)	SBP + 50 mmHg; sham 20 mmHg; 21.5 cm cuff width	IPC: 3 × 5 min. Test: SJFT Timing; applied immediately post-SJFT	↓ HR at 30 min *^,^^^ and 60 min *^,^^^^; ↑ CMJ at 60 min *^,^^^^; ↓ DOMS: 60 min *^,^^^	Post-exercise IPC may support cardiovascular and neuromuscular recovery between combat-sports bouts.

Abbreviations: AOP, arterial occlusion pressure; AR, active recovery; CMJ, countermovement jump; CON, control condition; DOMS, delayed onset muscle soreness; ES, effect size; F, female; M, male; IPC, ischemic preconditioning; min, minutes; NR, not reported; NMES, neuromuscular electrical stimulation; Pmax, maximal power; SBP, systolic blood pressure; SJFT, Special Judo Fitness Test; TT, time trial; TSI, tissue saturation index; VCO_2_, carbon dioxide output; VE, minute ventilation; VO_2_, oxygen uptake; VEGF, vascular endothelial growth factor; 1RM, one-repetition maximum; HR, heart rate; ↑, increase; ↓, decrease; *, *p* < 0.05 vs. control/sham; ^#^, small effect size vs. control; ^##^, moderate effect size vs. control; ^###^, large effect size vs. control; ^^^, small effect size; ^^^^, moderate effect size; ^^^^^, large effect size.

**Table 7:** IPC Guidelines for Performance and Recovery: Acute and Between-Bout Efforts.

Variable	Guidelines	Notes
Inflation Pressure	80–100% AOP or ~200–220 mmHg (cuff width dependent)	Sham pressures (20–40 mmHg) do not create the intended ischemic stimulus but may still produce placebo effects.
Cycles	3–4 cycles	Fewer cycles (e.g., 3) still produce performance effects; 4 cycles most common in research showing positive outcomes.
Inflation Duration	5-min per cycle	May provide sufficient stimulus to elicit endothelial responses and reperfusion-mediated vasodilatory responses.
Reperfusion Duration	5-min between cycles	Allows full reperfusion and metabolic clearance; supports nitric-oxide-related vasodilation.
Timing Relative to Exercise	Pre-event: Apply immediately before standard warm-up, leaving ~30–45 min prior to performance Recovery focus: immediately post-exercise	Preserves the standard warm-up sequence; repeat between bouts only where previously tolerated.
Application Context	Pre-competition performance priming, between sessions/competitive bouts, or post-exercise	Pre-performance IPC shows the most reliable ergogenic effects. Should be tested individually prior to implementation in a competition.
Restriction Method	Passive (no exercise during inflation); athlete seated or supine.	No exercise performed during inflation.
Cautions & Practical Notes	High individual variability—identify responders. Not a replacement for warm-up—integrate into existing pre-competition routine.	Use familiarization sessions and monitor inflation pressure, performance, and athlete feedback across repeated trials. Elite athletes generally respond more consistently than recreational athletes.

Abbreviations: AOP, arterial occlusion pressure; min, minutes; IPC, ischemic preconditioning.

**Table 8:** Guidelines for Ischemic Intra-Conditioning (IIC) During Training.

Variable	Guidelines	Notes
Inflation Pressure	60–80% AOP	Produces hypoxic/metabolic stress during rest while preserving the quality of work intervals. Start at 60% AOP and progress to 80% as tolerated.
Inflation Timing	Applied only during inter-repetition rest periods.	Distinguishes IIC from IPC. Deflate or remove BFR cuffs before each work interval begins.
Cycles and Occlusion Duration	1 cycle × 2.5–5 min per rest interval	Suited to high-intensity training with longer rest periods (≥3 min).
Training Duration	2–4 weeks (2–3 sessions/wk according to mesocycle goals)	Four-week interventions have increased VO_2max_ and molecular markers; longer training blocks may be required for performance transfer.
Application Context	Integrated within SIT/HIIT programs	Target aerobic and molecular adaptation while preserving work-bout quality.
Restriction Method	Passive, seated or supine.	No exercise performed during inflation.
Cautions and Practical Notes	Limited acute ergogenic effect; do not expect immediate performance improvement within single sessions.	Best applied for chronic adaptation rather than immediate performance gains. Requires multi-week commitment and should be integrated strategically within periodized training plans.

Abbreviations: AOP, arterial occlusion pressure; min, minutes; SIT, sprint interval training; HIIT, high-intensity interval training; IIC, ischemic intra-conditioning; BFR, blood flow restriction; VO_2max_, maximal oxygen uptake.

**Table 9:** BFR Guidelines for Pain Management.

Domain	Guidelines	Notes for Practice
Scope of Practice	S&C coaches do not diagnose or treat pathology	Diagnosis remains the responsibility of medical and physiotherapy professionals.
Medical Clearance	Mandatory prior to implementation	Required for athletes with pain, post-surgical status, or suspected pathology.
Interdisciplinary Communication	Ongoing collaboration required	Regular updates regarding symptoms, tolerance, and functional progression.
Applications	Chronic joint pain; tendinopathy; post-surgical rehabilitation; pain-limited maintenance; return-to-play transition; ongoing training	Only when medically cleared and integrated within a broader rehabilitation or performance plan.
Contraindications	Acute injury or inflammation; undiagnosed pain; worsening symptoms; acute rupture; joint instability (see Part 1: contraindications/safety screening)	Refer immediately if pain increases >2/10 from baseline, new neurological symptoms emerge, or systemic signs appear.
Initial Cuff Pressure	40–50% AOP	Progress conservatively based on tolerance and symptom response.
Pain Monitoring	Maintain ≤4/10 during exercise; ideally ≤2/10	Pain should remain stable or decrease across sessions.
Progression	Gradual increase in load, pressure, or volume	Only progress when pain remains stable or decreases over time.
Exercise Selection	Function-specific, sport-relevant movements	Align with return-to-play demands and physiotherapy progression.
Session Integration—Pain Modulation	May be applied daily or multiple times per day during short symptom-management phases	Used to attenuate pain, reduce movement-related discomfort, and support continued participation in training or rehabilitation.
Session Integration—Pre-Training Preparation	Applied immediately prior to skills, speed, or strength sessions	Used to reduce pain, increase tolerance to movement, and improve readiness for subsequent high-quality technical or physical training.
Session Integration—Training Stimulus	Integrated into resistance and selected cardiovascular sessions (2–3×·wk^−1^ or as dictated by training frequency)	Used to maintain or enhance strength and conditioning while limiting external mechanical load; redistributes rather than adds training stress.
Ongoing Review	Continuous reassessment required	Modify or cease BFR if symptoms worsen, tolerance declines, or recovery is impaired.

Abbreviations: S&C, strength and conditioning; AOP, arterial occlusion pressure; BFR, blood flow restriction; wk, week.

**Table 10:** BFR Guidelines for Bone Stress Injury and Bone Health.

Domain	Guidelines	Notes for Practice
Scope of Practice	Coaches do not diagnose or medically manage BSI, low BMD, or RED-S.	Medical oversight is required; align training decisions with clinician-defined restrictions and milestones.
Primary Purpose of BFR	Preserve muscle and conditioning while mechanical bone-loading activities are constrained	Direct bone benefits remain uncertain; muscle preservation supports re-loading readiness and reduces detraining.
Medical Clearance	Mandatory prior to implementation.	Confirm diagnosis, stage, permitted loading, and any vascular/neurological contraindications before commencing.
Interdisciplinary Communication	Maintain ongoing, documented coordination with the treating team.	Document and communicate training exposures, symptoms, tolerance, and functional progression.
Expectations	Position BFR as a bridging strategy, not a replacement for osteogenic loading.	BFR may support a pro-remodeling environment, but long-term bone outcomes still depend on energy availability, endocrine recovery, nutrition, and progressive weight-bearing loading.
Appropriate Contexts	Use during early-mid-BSI rehab; low BMD/RED-S recovery; later-stage fracture rehab; and in-season low-impact sports	Apply when impact/heavy loading is restricted; taper BFR reliance as conventional osteogenic loading is reintroduced.
Do Not Use or Pause BFR	Do not initiate or continue BFR without clearance or if symptoms worsen.	Pause and refer with escalating pain, swelling, night pain, neurological symptoms, or reduced function/tolerance across sessions.
Modes of Application	Select mode based on loading permission: passive BFR; contralateral training; low-load BFR-RE; selected intensity BFR-CV.	Use passive BFR for severe unloading/immobilization; contralateral training when one limb is restricted; low-load BFR-RE as the default when active exercise is allowed.
Exercise Selection and Loading	Progress from non-weight-bearing to partial to full weight-bearing as symptoms and clearance permit.	Match exercise selection to site-specific restrictions; progress complexity only when pain-free during and after sessions, with stable next-day tolerance.
Pre-Training Check	Confirm readiness each session (symptoms, function, restrictions, recovery status).	Document pain at rest and with key tasks, stiffness, swelling, sleep/recovery, and any new symptoms since the prior session.
Regression Criteria	Regress immediately with symptom recurrence or reduced tolerance.	Reduce load/volume/pressure and simplify movements if pain increases, function declines, or recovery worsens; pause BFR pending reassessment if red flags emerge.
Endurance and Low-Impact Sports	Use BFR to support strength and conditioning when impact is limited	Cyclists and swimmers may benefit from low-load BFR-RE as a musculoskeletal adjunct, but (non-BFR) impact-based and heavy resistance training remain essential for long-term bone optimization when appropriate.
Recurrence Prevention	Consider planned BFR exposure across the training week to complement key (non-BFR) high-intensity/technical sessions.	Repeated BFR exposure may support a favorable bone-turnover environment; direct effects in athletes remain unestablished.

Abbreviations: rehab, rehabilitation; BFR, blood flow restriction; BFR-RE, blood flow restriction resistance exercise; BFR-CV, blood flow restriction cardiovascular exercise; BSI, bone stress injury; BMD, bone mineral density; RED-S, relative energy deficiency in sport.

## Data Availability

No new data were generated or analyzed in this study. Data sharing is not applicable to this article.

## Abbreviations

The following abbreviations are used in this manuscript:

Abbreviation/Notation	Definition
a–v	Arteriovenous (difference)
AOP	Arterial occlusion pressure
BFR	Blood flow restriction
BFR-CV	Blood flow restriction cardiovascular exercise
BFR-RE	Blood flow restriction resistance exercise
BMD	Bone mineral density
BSI	Bone stress injury
CMJ	Countermovement jump
COD	Change of direction
CO	Cardiac output
CSA	Cross-sectional area
CTX	C-terminal telopeptide (bone resorption marker)
DOMS	Delayed onset muscle soreness
ES	Effect size
FMD	Flow-mediated dilation
HIF-1α	Hypoxia-inducible factor-1 alpha
HIIT	High-intensity interval training
HR	Heart rate
HRmax	Maximal heart rate
HRR	Heart rate reserve
IGF-1	Insulin-like growth factor 1
IIC	Ischemic intra-conditioning
IPC	Ischemic preconditioning
LOP	Limb occlusion pressure
MAP	Maximal aerobic power
MAS	Maximal aerobic speed
mRNA	Messenger RNA
MSS	Maximal sprint speed
MSTN	Myostatin
NMES	Neuromuscular electrical stimulation
P1NP	Procollagen type I N-terminal propeptide
PPO	Peak power output
RFD	Rate of force development
RED-S	Relative energy deficiency in sport
S&C	Strength and conditioning
SBP	Systolic blood pressure
SIT	Sprint interval training
SSG	Small-sided game
SV	Stroke volume
TSI	Tissue saturation index
TT	Time trial
VCO_2_	Carbon dioxide output
VE	Minute ventilation
VEmax	Maximal minute ventilation
VEGF	Vascular endothelial growth factor
VO_2_	Oxygen uptake
VO_2max_	Maximal oxygen uptake
VO_2peak_	Peak oxygen uptake
